# Wellbeing in the Republic of Macedonia

**DOI:** 10.1080/17571472.2017.1409955

**Published:** 2017-12-18

**Authors:** Nelio Oliveira

**Affiliations:** Economist

## Why this matters to me

I have always been intrigued by the Balkans. Then, I became friendly with a Macedonian family that came to the UK about 10 years ago. Theyset up a small transport business from a small flat in London’s periphery. They inspired me to get to know their cultural roots and visit the country.

## Key message

The well being of Macedonia depends on diversifying production away from wine, creating jobs for the young population.

## Wellbeing in the Republic of Macedonia

The Republic of Macedonia is the smallest, poorest and most vulnerable country in the Balkans. It is a landlocked country, one of the successor states of the former Yugoslavia, from which it declared independence in 1991. It has borders with Kosovo to the northwest, Serbia to the north, Bulgaria to the east, Greece to the south, and Albania to the west – countries that have some kind of claim to its territory, culture and even its name. It isn’t in the European Union.

The Kingdom of Macedonia was established some time during the 7th Century BC. Macedonian dominance of Greece was secured by Philip II and subsequently by his son Alexander ‘The Great’. Alexander was born in 356 BC in Pella, the ancient capital of Macedonia, situated now in the Greek part of Macedonia. From Macedonia he conquered territories all the way to India, including Persia, opening out a corridor for East-West discourse and trade [1].

After several wars against the Macedonians, the Romans defeated them in 146 BC and the country became part of the Roman Empire. Its vast arable land and rich pastures allowed huge fortunes to be amassed by the Roman rulersin a society based on slave labour. The centre of Roman power was transferred to Byzantium in the 4th Century AD and since then various armies have controlled the country, includingBulgarians, Serbs and the Ottoman empire (13th–19th centuries) [2].

The Republic of Macedonia is a pristine land with mountains, lakes and old cities. It has the economic, cultural and physical conditions to offer a good quality of life to its small population of about 2.1 million people. Well-being has decreased since the breakdown of the former Yugoslavia. This is due mostly to the de-industrialization process, and weak political institutions in the country.

The climate and land are very favourable to agriculture. Most of the country enjoys a Mediterranean climate as, despite being a landlocked country, it is near to the Mediterranean Sea. This provides high quality land, varied food, excellent wine, good weather, and low pollution. However Skopje, where half of the population lives, has an unhealthy summer because it is situated in a valley that concentrates the pollution created by the vehicles. Despite this, it is worth a visit for the abundance of new gigantic bronze statues of ancient heroes, museums and excellent food and wines.

Luckily, it still keeps a strong educational sector and the cultural scene is dynamic with several good museums in the capital. The population strongly and peacefully participates in the difficult political life of the country. The health sector needs improvements, as will be shown further in this article.

Life expectancy at birth had increased from 58 years in 1960 to 76 in 2015. Income per capita has changed from US$ 1330 in 1994 to US$ 4980 in 2016. However, growth slowed to 2.4% in 2016 and turned negative in the first half of 2017, as political uncertainty affected investment. The unemployment rate fell to 22.8% in the first half of 2017, a historic low. Youth and long-term unemployment remain high at 46 and 81%, respectively [3].

Macedonia experienced a serious political crisis in 2014–2017 due to disputes between the main political parties. In the view of Nade Proeva, the main source of the dispute is that ‘Macedonian politicians of Albanian origin are systematically denying the natural and constitutional name of the nation and the state they live in’ [4]. The Albanian minority represents about 15% of the population, living in the western border with Albania.

Although the Albanian minority is recognised by the Constitution, including the use of their language, manyAlbanians believe in the idea of a ‘Greater Albania’ (by integrating the Western party of Macedoniawith Albania) and this may be behind the dispute. Additionally, Greece does not recognise ‘Macedonia’ as the name of the Republic; they instead would like to add a geographical addition, like Northern Macedonian Republic [5]. Both disputes have become international issues involving the USA, EU and Russia.

An agreement between the Macedonian parties resulted in elections in December 2016, and the formation of a new Government in June 2017. The Government adopted a Plan that includes a set of measures that will be implemented in the next three, six, and nine months to accelerate the process of EU and NATO accession. It is expected thatEU accession negotiations will start in Spring 2018 [3].

The Constitution of the Republic of Macedonia guarantees universal access to healthcare for all citizens. Compulsory insurance coverage encompasses nearly the entire population. This enables the provision of health care services and allowances. The country has gone through a number of healthcare reforms in recent years. The quality of medical care declined after the breakdown of former Yugoslavia and adoption of the market economy. Macedonia is still characterized by relatively high birth rates [6].

Overall, the health situation in the Republic of Macedonia is characterized by a high representation of non-communicable diseases in morbidity and mortality, which is typical for developed countries. The aetiology of non-communicable diseases is related to lifestyle. The population, especially in the capital, are facing the challenges of bad lifestyle habits, such as: fatty diet, smoking, excessive use of alcohol and other psychoactive substances, leading to addiction, infectious diseases, physical and mental health disorders [6].

A 2013 review of the public health care sector in the Republic of Macedonia concluded that the Macedonians consider the public healthcare system to be medium-good in all aspects: accessibility, availability, quality of health care services and population’s confidence. There are a substantial number of hospital beds in highly specialized hospitals. From 175 people interviewed nationally, 125 believe that state-of-the-art treatment exist all over the country (‘yes’: 33.6% and ‘rather yes’: 44.8%) [6].

They believe that the services are accessible to everybody, free of major charges (‘yes’: 31.2% and ‘rather yes’: 45.6%). The 50 interviewees who did not agree that state-of-the-art treatment exists throughout the country pointed to lack of pharmacies and proper medicines in rural areas, with a gap between the availability and quality of services in rural vs. urban areas [6].

Despite the health issues mentioned above, the great challenge for the country is the unstable political situation and role of ethnical minorities, especially Albanians, as explained above. This situation makes the country vulnerable and isolated on the international scene.

As explains Professor Proeva:

The proclamation of the sovereignty of Macedonia in 1991 provoked ‘ardent’ denying of its name, language, nation or a part of its territory. Therefore, these issues were politicized in a rather vulgar manner. Journalists, politicians, intellectuals and even certain researchers, first of all archeologists, started to create new national myth allegedly aimed to defend the name of the nation. [4]

In conclusion, the quality of the health sector has declined after the disintegration of Yugoslavia. However, the public healthcare system still scores medium-good in all aspects – availability, quality of health care services and popular confidence. The population in the street, in the capital and interior, looks robust and healthy.However, to improve the social-economic sectors, the Macedonians need to diversify its production from a wine economy and address the country’s political-ethnical situation and its insertion in the European space.
